# Evaluating the Role of Objective Structured Clinical Examination as a Summative Assessment Tool in Undergraduate and Postgraduate Psychiatry Residents

**DOI:** 10.7759/cureus.67640

**Published:** 2024-08-23

**Authors:** Abhishek Pathak, Vimala Venkatesh, Anjoo Agarwal, Jyoti Chopra

**Affiliations:** 1 Psychiatry, Hind Institute of Medical Sciences, Sitapur, IND; 2 Microbiology, King George's Medical University, Lucknow, IND; 3 Obstetrics and Gynaecology, King George's Medical University, Lucknow, IND; 4 Anatomy, King George's Medical University, Lucknow, IND

**Keywords:** validity, reliability, faculty perception, student perception, assessment, competence, medical education, psychiatry, objective structured clinical examination

## Abstract

Background: The Objective Structured Clinical Examination (OSCE) is the gold standard and universal format to assess medical students' clinical competence in a comprehensive, reliable, and valid manner. OSCE is gaining global popularity for assessing medical students in various specialties. Our country uses it in multiple disciplines, but its application in psychiatry remains limited. OSCE is a resource-demanding assessment method that can face numerous challenges. A comprehensive assessment of perceptions regarding OSCE can help identify areas that need improvement. Therefore, this study was conducted to assess the perceptions of students and examiners toward OSCE in psychiatry.

Aims and objectives: This study aims to evaluate the role of Objective Structured Clinical Examination as a summative assessment tool in assessing competency in undergraduate (as part of their ward leaving examinations in psychiatry) and postgraduate psychiatry residents (as part of their six-monthly assessments) and analyze the perceptions of students as well as of the faculty members regarding OSCE.

Methods: Six MD students and 49 MBBS students took the OSCE as part of their ward-level exams in psychiatry. In the presence of four faculty members of the psychiatry department, students completed their six-month summative exams. The OSCE was held at separate times for UG and PG students. UG and PG students utilized different stations (PG students had a harder level). A 10-item questionnaire was given to examiners and students at the end of the OSCE to get their opinions regarding the OSCE. Furthermore, data gathered from the faculty through an open-ended questionnaire was compiled and displayed thematically. Since the Likert scale survey generated ordinal data, the statistical analysis was conducted using the median, interquartile range (IQR), and chi-square test. The chi-square test was used to compare the variables. A P-value of less than 0.05 was deemed statistically noteworthy.

Results: Four faculty members and fifty-five students in all answered the questionnaire. Regarding the OSCE's characteristics, validity, reliability, and transparency, the majority of students expressed positive opinions. In a similar vein, most examiners had positive perceptions pertaining to OSCE's administration, structure, and procedures. Certain areas, such as "improved confidence in teaching clinical skills" and "improved confidence in giving students structured feedback," were also emphasized by thematic analysis of faculty members.

Conclusion: In general, both students and examiners had extremely favorable perceptions of and embraced the OSCE. Improved faculty orientation and student preparation for the OSCE may help allay anxiety and overcome hesitation related to the exam.

## Introduction

Developed in the 1970s, the Objective Structured Clinical Examination (OSCE) is regarded as the benchmark for evaluating clinical competence in medical students and is used worldwide [1,2]. The OSCE assesses skills and abilities at the "show-how" level to mirror actual clinical performance [3]. Recently, India transitioned from a knowledge-based medical education system centered on competency to one that prioritized practical application in real-life clinical scenarios. OSCEs are gradually being adopted to assess both undergraduate and postgraduate skills. These structured exams tackle problems encountered in traditional long-case clinical assessments, including issues with transparency, inconsistent scoring, subjectivity, and a limited range of real-life cases. [4-6].

The goal of competency-based medical education is to fairly and objectively assess students’ performance. As undergraduate and postgraduate curricula evolve, structured and objective assessment methods at higher skill levels are the need of the hour [7-10]. Effective assessment should be goal-oriented and involve multidimensional integrated learning, ensuring alignment of educational performance with set objectives. Traditional viva assessments face problems with content validity, reliability, inconsistent grading, and a lack of standardization. OSCEs, however, support the National Medical Commission’s (NMC) vision by evaluating knowledge, skills, and attitudes. They promote a deeper grasp of subjects by focusing on practical application rather than rote memorization. OSCEs provide an unbiased evaluation, using the same competency checklist for all students. As both undergraduate and postgraduate curricula advance, there is an increasing need for more structured and objective assessment methods at higher skill levels [9-10].

Although research on OSCEs in psychiatry exams is scarce, they are gaining prominence in Indian medical colleges, especially during the pandemic when patient inflow was relatively low [11].

In India, OSCEs are still in their infancy. Research indicates a limited correlation between medical school success and performance in residency, even though undergraduate medical education is a crucial stepping stone to postgraduate studies and medical careers [12]. Considering these limitations, the Department of Psychiatry, HIMS, Sitapur, attempted to introduce OSCE for undergraduates as a part of the ward leaving exam and six monthly assessments for postgraduate psychiatry residents.

## Materials and methods

Study design

This is a cross-sectional observational and perception analysis study.

Participants

This study involved MBBS UG students posted in the Department of Psychiatry and Postgraduate Psychiatry Residents. The faculty members teaching the subject and involved in the monitoring of observed stations were also part of the study.

Sampling and measures

All the MBBS students posted in the Department of Psychiatry during their end-posting assessment and all the Postgraduate Psychiatry Residents, as part of their six-monthly assessments, were also included in the study. OSCE for UG and PG students was conducted at different points in time. Different stations were used for UG and PG students (the level was tougher for PG students). All six PG students were subjected to the same OSCE stations. Any student who was absent on the day of the assessment was excluded.

Ethical considerations

The Institutional Ethics Committee gave ethical clearance for the research, which was carried out under reference number IHEC-HIMSA/FA/RD-05/4-24.

Study protocol

This protocol involves six OSCE stations designed to assess various clinical skills: (a) assessment of phenomenology; (b) demonstration of a mental status examination; (c) assessment using a relevant scale; (d) breaking bad news using the spikes model; (e) psychopharmacology; (f) spotters like neuroimaging, EEG, and psychological tests; and (g) managing a crisis.

Stations were required to be completed in ten minutes, with each station providing a brief introduction to the clinical scenario and the task. A two-minute break was allotted between stations. Examiners used an objective checklist developed by the psychiatry department to grade the students. Each station was allotted 10 marks. After the conclusion of the examinations, students and faculty were interviewed to discuss their experiences.

After the OSCE, students' perceptions of the OSCE as a clinical evaluation method were gathered in the classroom on the same day using a 10-item questionnaire adapted from the OSCE evaluation tool by Pierre et al. [13]. This standardized questionnaire is valid and reliable (0.82), available in the public domain, and does not require special permission for use. The questionnaire, based on previous studies and employing a five-point Likert scale, underwent a few modifications. To ensure its validity, it was reviewed by medical education faculty and a scientific committee to confirm that it effectively measured the intended aspects.

Data collection

Participants (students and examiners) were requested to complete a questionnaire after the OSCE to gather data for the current research. The questionnaire's design ensured participant anonymity and confidentiality of the information collected. The questionnaire had a printed page attached with a short explanation of the study and a permission request. Participants in the research gave their signatures on the informed consent form and completed the questionnaire. Any collected identifying information was detached from the response data and substituted with codes. Only the research team had access to the key linking these codes to identities, ensuring data confidentiality. All candidates completed this procedure on-site at the end of the OSCE.

Data analysis

The information gathered was both qualitative and quantitative. We imported the data into the Statistical Package for the Social Sciences (SPSS) version 23.0 from IBM Corporation, Armonk, NY, USA. The OSCE mean and standard deviation were computed using the students' age and grade data. The dependability of the instruments was determined by calculating Cronbach's alpha. In particular, whether the distribution of response frequencies in each item was different enough to reject the null hypothesis that the distribution was due to chance was determined using percentages, median, IQR, and chi-squared analysis with Yates' correction. P ≤0.05 served as the crucial value for rejecting the null hypothesis. The data gathered from open-ended inquiries was compiled and organized thematically. The feasibility of applying the observed station as a regular evaluation technique was evaluated by reviewing the replies to the open-ended questionnaire based on a paper survey. To facilitate comprehension, the responses were divided into topics.

## Results

The demographic data from Table 1 reveals several insights into the characteristics and performance of the respondents. The gender distribution indicates a higher proportion of male students ‘36 (66%)’ compared to female students ‘19 (34%)’. Age-wise, the majority of students fall within the 19- to 22-year age group ‘22 (40%)’, followed by ‘18 (32.72%)’ in the 23- to 26-year range, and ‘15 (27.72%)’ aged 26 and above. Most students were in their second year of MBBS ‘49 (90%)’. Regarding OSCE scores, a significant majority of ‘41 (74.54%)’ scored above 30 out of 60, indicating good performance, while ‘14 (25.45%)’ scored below 30. The gender distribution showed male predominance and postgraduate students were a minority.

**Table 1:** Demographic characteristics of respondents (n=55)

Categories		Frequency (%)
Gender	Male	36 (65.45)
	Female	19 (34.54)
Age group	From 19 to 22	22 (40)
	From 23 to 26	18 (32.72)
	26 and above	15 (27.72)
Study year	MBBS - 2nd year	49 (89.09)
	PG - JR 1	2 (3.63)
	PG - JR 2	2 (3.63)
	PG - JR 3	2 (3.63)
Score (out of 60)	Below 30	14 (25.45)
	Above 30	41 (74.54)

**Figure 1:**
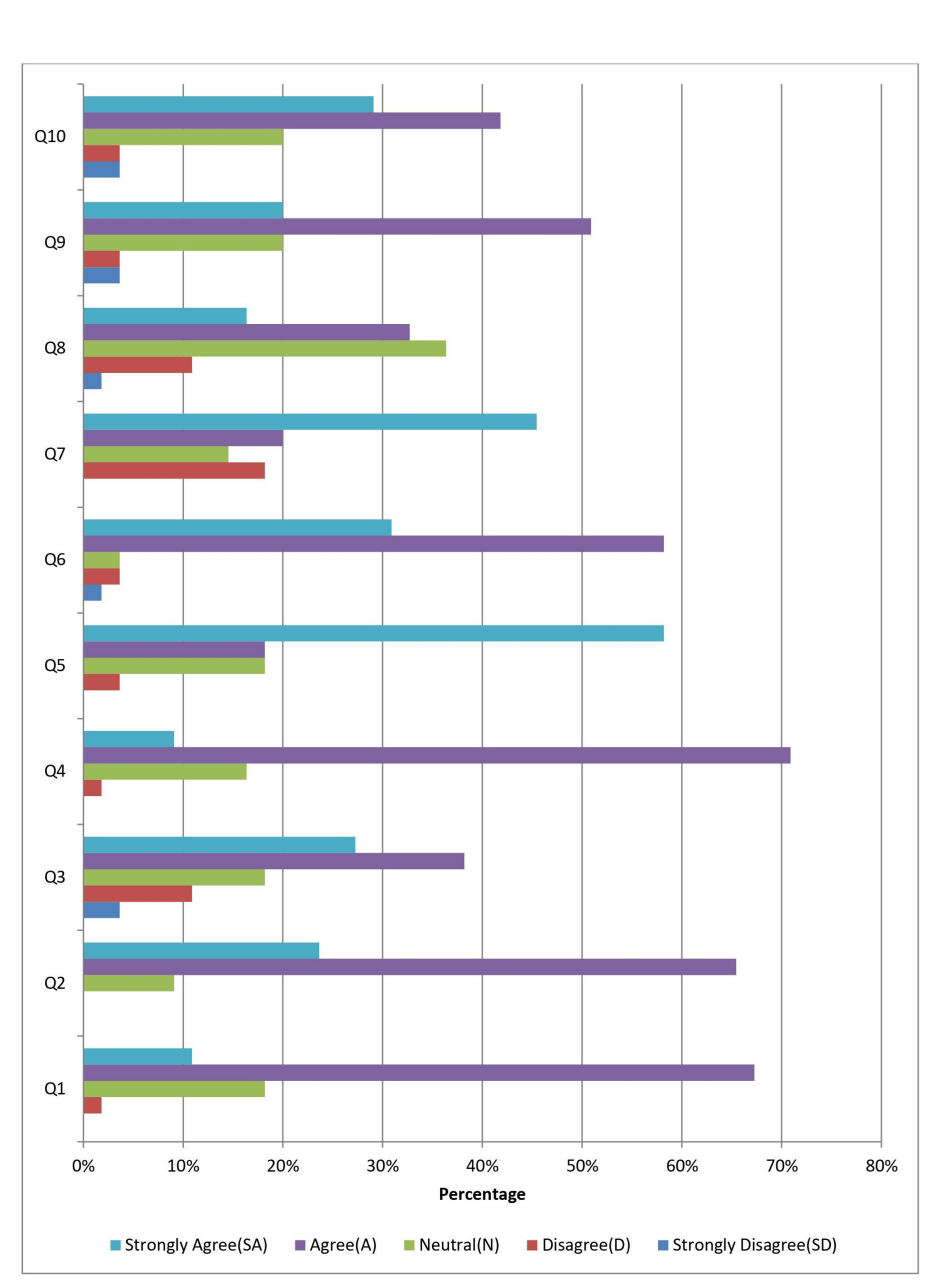
OSCE Feedback Questions and Scores (frequency (%) out of 55) OSCE: objective structured clinical examination

As shown in Table 2 and Figure 1, the OSCE feedback data show that most students '43 (78.17%)' found the questions were appropriately framed. A significant majority of '49 (89.08%)' felt the scoring was fair across different observers. '36 (65.45%)' believed attending demonstrations helped improve scores, though '8 (14.53%)' disagreed. The majority, '44 (80%)', viewed the stations as more skill-based than viva voce, and '42 (76.36%)' felt compelled to change their learning methods. Most students '49 (89.08%)' believed they would retain the skills longer. However, only '27 (49.08%)' felt the stations were unbiased, with '20 (36.36%)' being neutral. Anxiety was perceived to be higher in viva voce '39 (70.9%)', and '39 (70.9%)' felt the time allotted was sufficient.

**Table 2:** OSCE Feedback Questions and Scores (frequency (%) out of 55) OSCE: objective structured clinical examination

S.NO.	OSCE feedback questions	Strongly disagree (SD)	Disagree (D)	Neutral (N)	Agree (A)	Strongly agree (SA)
1	Were the questions framed appropriately?	0 (0%)	1 (1.81%)	10 (18.18%)	37 (67.27%)	6 (10.90%)
2	Do you think the score would be the same no matter who the observer is?	0 (0%)	0 (0%)	5 (9.09%)	36 (65.45%)	13 (23.63%)
3	Do you feel attending the clinics properly would help in increasing the score?	2 (3.63%)	6 (10.90%)	10 (18.18%)	21 (38.18%)	15 (27.27%)
4	Do you feel these observed stations were more skill-based than viva voce?	0 (0%)	1 (1.81%)	9 (16.36%)	39 (70.90%)	5 (9.09%)
5	Did these stations compel you to change your method of learning?	0 (0%)	2 (3.63%)	10 (18.18%)	10 (18.18%)	32 (58.18%)
6	Do you think you will remember the skill asked for a longer time?	1 (1.81%)	2 (3.63%)	2 (3.63%)	32 (58.18%)	17 (30.90%)
7	Were the questions asked easy to understand?	0 (0%)	10 (18.18%)	8 (14.54%)	11 (20%)	25 (45.45%)
8	Do you feel that these stations are unbiased?	1 (1.81%)	6 (10.90%)	20 (36.36%)	18 (32.72%)	9 (16.36%)
9	Do you feel that the level of student’s anxiety is more in viva voce as compared to observed stations?	2 (3.63%)	2 (3.63%)	11 (20%)	28 (50.90%)	11 (20%)
10	Was the time provided enough for the observed stations?	2 (3.63%)	2 (3.63%)	11 (20%)	23 (41.81%)	16 (29.09%)

Table 3 depicts a comparison of OSCE domain perceptions between male and female students, revealing interesting insights into their varied experiences and perceptions within the assessment framework. Across the ten domains surveyed, significant differences emerged in several key areas. First, male students rated the appropriateness of framed questions significantly higher than their female counterparts (2.61 ± 0.599 vs. 2.26 ± 0.653, p = 0.026), indicating a more favorable perception of question clarity among males. Similarly, male students felt more strongly that attending demonstrations would enhance their scores compared to females (2.22 ± 0.929 vs. 1.53 ± 0.772, p = 0.003), highlighting a disparity in perceived benefits from instructional sessions.

**Table 3:** OSCE domains comparison between male and female respondent OSCE: objective structured clinical examination

S.NO.	OSCE domains	Mean ± SD		Mean difference	t-value	p-value
		Male	Female			
1	Were the questions framed appropriately?	2.61 ± 0.599	2.26 ± 0.653	0.35	2.314	0.026*
2	Do you think the score would be the same no matter who the observer is?	2.81 ± 0.401	2.63 ± 0.496	0.18	1.792	0.083
3	Do you feel attending the demonstrations properly would help in increasing the scores?	2.22 ± 0.929	1.53 ± 0.772	0.69	3.141	0.003*
4	Do you feel these observed stations were more skill-based than viva voce?	2.69 ± 0.525	2.42 ± 0.607	0.27	1.631	0.113
5	Did these stations compel you to change your method of learning?	3.44 ± 0.843	2.95 ± 0.911	0.49	1.942	0.057*
6	Do you think you will remember the skills asked for a longer time?	2.58 ± 0.732	2.21 ± 0.855	0.37	1.784	0.082
7	Were the questions asked easy to understand?	1.94 ± 0.674	1.53 ± 0.513	0.41	2.621	0.012*
8	Do you feel that these stations are unbiased?	2.28 ± 0.849	1.63 ± 0.684	0.65	3.071	0.004*
9	Do you feel that the level of student anxiety is higher in viva voce as compared to the observed station?	2.42 ± 0.841	1.89 ± 0.875	0.53	2.123	0.041*
10	Was the time provided enough for the observed stations?	2.42 ± 0.841	1.89 ± 0.875	0.53	2.123	0.041*

Conversely, no significant differences were observed in perceptions regarding whether observed stations were more skill-based than viva voce examinations, with both genders showing similar mean scores (2.69 ± 0.525 for males and 2.42 ± 0.607 for females, p = 0.113). Likewise, there was no statistical difference in the perception of whether stations influenced a change in learning methods between male and female students (3.44 ± 0.843 vs. 2.95 ± 0.911, p = 0.057), indicating comparable impacts on learning approach adaptation.

Furthermore, male students generally found the questions easier to understand (1.94 ± 0.674 vs. 1.53 ± 0.513, p = 0.012) and perceived stations to be more unbiased (2.28 ± 0.849 vs. 1.63 ± 0.684, p = 0.004) compared to female students. Additionally, males reported higher anxiety levels during viva voce compared to observed stations (2.42 ± 0.841 vs. 1.89 ± 0.875, p = 0.041), suggesting varying stress responses across assessment formats.

While male and female students generally share similar perceptions in certain domains of the OSCE assessment, significant gender disparities exist in how they perceive question clarity, instructional benefits, ease of understanding, perceived bias, and anxiety levels. These findings underscore the importance of considering gender-specific factors in assessment design and supporting strategies aimed at mitigating perceived biases and enhancing the overall assessment experience and fairness.

In Table 4, the OSCE stations reveal nuanced perceptions among respondents. Overall, participants agreed that questions were appropriately framed (M = 2.7 ± 0.8) and recognized the skill-based nature of the stations compared to viva voce (M = 2.7 ± 0.7). Demonstrations were viewed positively for improving scores (M = 2.3 ± 0.8), and stations significantly influenced changes in learning methods (M = 3.2 ± 0.9). However, concerns were raised by some students about question clarity (M = 1.9 ± 0.8) and potential bias (M = 2.2 ± 1.1), with higher anxiety levels perceived in viva voce settings (M = 2.3 ± 1.0). These insights underscore areas for improvement in question formulation and bias mitigation while affirming the effectiveness of OSCE in enhancing skill retention and educational methods.

**Table 4:** Analysis of questionnaire responses

S.NO.	OSCE items	Mean ± SD	Chi-square (p-value)
1	Were the questions framed appropriately?	2.7±0.8	21.55 (0.00024)
2	Do you think the score would be the same no matter who the observer is?	2.7±0.7	21.55 (0.00024)
3	Do you feel attending the demonstration properly would help in increasing the score?	2.3±0.8	30.8 (0.0000015)
4	Do you feel these observed stations were more skill-based than viva voce?	2.7±0.7	30.8 (0.0000015)
5	Did these stations compel you to change your method of learning?	3.2±0.9	43.4 (0.0000000014)
6	Do you think you will remember the skill asked for a longer time?	2.6±0.9	26.3 (0.00001)
7	Were the questions asked easy to understand?	1.9±0.8	21.2 (0.00028)
8	Do you feel that these stations are unbiased?	2.2±1.1	23.75 (0.00006)
9	Do you feel that the level of student’s anxiety is more in viva voce as compared to observed stations?	2.3±1.0	30.2 (0.0000023)
10	Was the time provided enough for the observed stations?	2.3±1.0	23.55 (0.00006)
Total		2.4±0.5	-

Table 5 presents the summarized responses from faculty members regarding their perceptions of the observed stations used for assessment, covering various aspects such as validity, reliability, educational impact, and student anxiety. First, a significant majority, '3 (75%)' of faculty members, strongly agree that observed stations are a valid method of assessment, indicating widespread confidence in their effectiveness for evaluating student performance. Conversely, a substantial portion of '3 (75%)' also express disagreement or strong disagreement regarding the reliability of these stations for assessing practical skills, suggesting concerns about the consistency and dependability of the assessment outcomes. Furthermore, there is unanimous agreement '4 (100%)' among faculty members that attending demonstrations properly would enhance student scores, underscoring the perceived importance of preparation and engagement in improving academic performance. Regarding the impact on teaching-learning methods, responses were evenly divided (25% each) on whether the observed stations prompted modifications in teaching methods. This reflects varied perceptions of the stations’ influence on pedagogical approaches.

**Table 5:** Responses of questionnaire for faculty on observed stations

S.No	Questions	Strongly disagree	Disagree	Neutral	Agree	Strongly agree
1	Do you feel the observed stations are a valid method of assessment?	-	-	-	1 (25%)	3 (75%)
2	Do you feel this method is a reliable tool for assessing practical skills?	-	-	-	3 (75%)	1 (25%)
3	Do you feel attending the demonstrations properly would help in increasing the scores?	-	-	-	-	4 (100%)
4	Do you feel these observed stations were more skill-based than viva voce?	-	-	-	1 (25%)	3 (75%)
5	Did these stations lead you to modify the way you teach?	1 (25%)	1 (25%)	1 (25%)	1 (25%)	-
6	Do you think students will remember the skills asked for a longer time?	-	-	-	2 (50%)	2 (50%)
7	Were the stations difficult to create?	1 (25%)	2 (50%)	-	1 (25%)	-
8	Do you feel that these stations are unbiased?	-	1 (25%)	-	-	3 (75%)
9	Do you feel that the level of student anxiety is higher in viva voce compared to observed stations?	-	1 (25%)	2 (50%)	-	1 (25%)
10	Do you recommend continuing this method for assessment?	-	-	-	1 (25%)	3 (75%)

Regarding student retention of skills, half of the faculty members '2 (50%)' believe that students will remember the skills assessed through observed stations for a longer duration, indicating moderate confidence in the lasting educational impact of these assessments. In terms of operational challenges, half of the respondents '2 (50%)' find creating stations somewhat or very difficult, highlighting logistical hurdles in implementing observed stations effectively. Moreover, a significant majority, '3 (75%)', perceive observed stations as unbiased in their assessment approach, suggesting confidence in the fairness and impartiality of the evaluation process. However, opinions diverge regarding student anxiety levels, with '2 (50%)' feeling that anxiety is higher during viva voce examinations compared to observed stations, while '1 (25%)' perceives the opposite. An overwhelming majority of '3 (75%)' faculty members recommend continuing utilization of observed stations for assessment purposes, indicating strong overall support for integrating these stations into the assessment framework. These findings provide valuable insights into faculty perspectives on observed stations, highlighting strengths along with areas for potential improvement in their implementation and perceived educational impact.

The list of open-ended questionnaires was given to the faculty, and they all agreed that the observed stations were a valid and reliable method for assessing skills and application-based knowledge in psychiatry. They also believed that “in anticipation of a more structured form of skill-based practical examination, the student’s day-to-day study sessions focused on practical and applied knowledge of theoretical concepts. This change has been facilitated by small group teaching with a more informed approach and group discussions among students. 'On the matter of feasibility, the faculty believed that creating an observed station requires pre-planning well before a scheduled practical exam. Due to the various components involved in setting up such a station, departmental cooperation among faculty and between faculty and staff is necessary and promotes teamwork.' Creating such stations also requires understanding the practical competencies that need to be assessed in psychiatry.

## Discussion

We initially used traditional assessment methods for both formative and summative evaluations, such as short and long cases and viva voce. This approach stemmed from our experiences as students and our subsequent comfort with these conventional methods as faculty. However, recognizing the limitations of this traditional system and the concerns expressed by our students over time, we began exploring alternative assessment methods. We concentrated on the OSCE because it addresses some key shortcomings of traditional evaluation methods. Since its inception about 40 years ago, this assessment tool has proven its credibility and is currently utilized at graduate and postgraduate levels across various medical disciplines worldwide.

Initially, when the OSCE was introduced, medical students engaged with simulated patients at different stations, focusing on tasks such as history-taking, performing physical exams, providing counseling, and patient management [14,15]. Over time, the OSCE has evolved to include assessments of communication skills. Nowadays, leading medical schools in the US, UK, Canada, and elsewhere regularly use the OSCE alongside traditional cognitive assessments, such as essays and multiple-choice tests, to evaluate clinical competence and skills [16,17].

Students’ perceptions of the OSCE

The majority of students (78.17%) thought the questions had a good framework. A vast majority of respondents (89.08%) believed that the scoring was equitable among various observers. Furthermore, most students considered the OSCE a legitimate, unbiased, and trustworthy evaluation. This impression can be explained by the fact that every student is assessed using the same standardized patient, and each station has the same set of questions and checklists meant to reduce bias. Additionally, 76.36% of students were inspired to alter their study strategies, and 80% of students thought the stations placed a greater emphasis on psychomotor skills compared to traditional viva-voce examinations. Students also believed that OSCE results were a reliable indicator of fundamental clinical skills, independent of character attributes or interpersonal connections. Alkhathlan et al., in assessing the perspectives of surgical students on the accuracy and fairness of the OSCE at Dow University of Health Sciences, reached a comparable conclusion. According to his findings, most students thought the OSCE was a fair assessment method [18].

Furthermore, compared to their final test results, students believed that OSCE was a more relevant and trustworthy indicator of their clinical competence [19]. This is probably due to the OSCE's strict time constraints and meticulous design, which systematically covers every subject area and a wide range of domains [20,21]. The widespread agreement among students that the OSCE provided a fair assessment experience can be explained by the fact that standardized scenarios, well-prepared standardized patients, and consistent evaluation checklists are integral components of OSCE.

Unlike the study by Alkhathlan et al. [18], which examined the perceptions of male and female medical students regarding the effectiveness of the OSCE at King Saud bin Abdulaziz University for Health Sciences, the results indicated that only about one-third of students felt that OSCE scores genuinely reflected crucial clinical skills. Additionally, around half of the students thought personality and social interactions might influence OSCE scores. Mitchell et al. highlighted the importance of combining OSCE with other assessment methods to achieve accurate and comprehensive evaluations of medical students' clinical abilities [19].

According to the study's findings, students were more satisfied with the OSCE than with traditional viva voce examinations. They were more inclined to favor the OSCE compared to the conventional examinations. These findings are consistent with other studies that emphasized the benefits of using OSCE as an assessment tool. The favorable view of the OSCE can be ascribed to how well it aligns with course goals, its contribution to enhancing instruction, its ability to sync theoretical knowledge with practical application, its role in improving decision-making skills, and its refinement of assessment methods [20-23].

Huang et al. found that students who scored higher on the OSCE assessment method showed more self-confidence in clinical practice exams [24], which is in contrast to the findings of Rasoulian et al. that students’ dissatisfaction with OSCE has been attributed to the artificial nature of the environment and the use of simulated patients [23].

In contrast to conventional viva voce tests, students in the present research felt that the OSCE was less demanding, scary, and frightening. This impression might be connected to the OSCE's perceived impartiality. Students taking the OSCE are also better aware of the format and the particular knowledge needed for it. The present results, however, are at odds with those of Brosnan et al., who said that more than half of their students thought the OSCE was more stressful than previous official exams. The OSCE, or Structured Clinical Examination, is a taxing evaluation. The novelty of the evaluation structure was often blamed for stressful OSCE experiences [20,21].

The results of this study show that students thought the OSCE allotted time for each station was enough. This contrasts with the findings of Bayomi and Yousri, who found that students wanted longer test times [25]. Troncon also highlighted that students had trouble managing their time throughout the OSCE [26]. Immaturity and a lack of specialized training in time management strategies might be the causes of these issues. Awaisu et al. also reported dissatisfaction with the allotted time at each station, emphasizing the difficulty of assigning distinct time restrictions to different OSCE sites [27].

There were notable gender variations regarding participants' satisfaction with the Objective Structured Clinical Examination (p<0.05). First and foremost, compared to their female peers, male students rated the appropriateness of the questions much higher, suggesting they had a more positive opinion regarding the clarity of the questions. Additionally, male students found the stations to be less biased and the questions easier to understand compared to female students. In addition, male students expressed greater anxiety during viva voce examinations than at observed stations, indicating that stress reactions varied throughout assessment modalities.

While the opinions of male and female students were comparable in several areas of the OSCE evaluation, there were notable differences between the genders regarding perceptions of bias, anxiety, ease of understanding, instructional advantages, and question clarity. These results emphasize the importance of considering gender when designing assessments and implementing support mechanisms to reduce perceived biases, improve assessment fairness, and enhance the overall OSCE experience.

The results for UG and PG students were combined due to the small number of PG students. It was not possible to perform a statistical comparison of their perceptions because of the disparity in sample sizes. This imbalance may impact the generalizability and dependability of the findings. Due to the small sample size of postgraduate students, statistical power is decreased, making it more difficult to identify significant differences and draw firm conclusions. Compared to UG students, postgraduate students had a more positive opinion of the OSCE (better understanding of the OSCE stations, better time management, and less anxiety). This may be explained by the fact that PG students have more advanced clinical knowledge and competence and are better adapted to this format of assessment. Their increased proficiency might boost their confidence while completing OSCE activities, resulting in an enhanced positive perception of the OSCE. However, these findings should be considered cautiously due to possible statistical limitations.

Faculty’s perceptions of the OSCE

The feedback questionnaire designed to evaluate faculty perceptions of the OSCE sessions revealed that all faculty members (100%) either agreed or strongly agreed that the observed station is a valid and reliable way to assess practical skills. These findings are consistent with research conducted by Brazeau et al., in which the faculty members said that the process of providing feedback to the students was educationally fulfilling [28].

The faculty members were evenly split on whether these stations led them to modify their teaching-learning methods, with 25% each strongly disagreeing, agreeing, and strongly agreeing. This might be the case, as some of them may have already placed greater focus on practical applications and shown how certain competencies work, while others may have adjusted their teaching methods to account for changes in the assessment methods. All the faculty members (100%) agreed or strongly agreed that students would retain the necessary abilities for a longer period of time. The majority of faculty members (75%) agreed or strongly agreed that student anxiety is higher in viva voce as compared to OSCE. This is possible as the faculty take viva, and they might appear intimidating to the students. Most faculty members (100%) agreed or strongly agreed to recommend continuing this method for assessment.

The validity and reliability of the assessment process depend on a sufficient and representative sample of clinical tasks and direct performance observation, as highlighted by Sood and Singh [29]. Our current study supports supplementing the conventional assessment method with that of observed stations. According to Singh, a well-designed and administered OSCE may provide a plethora of data on students' clinical competency [30].

Kordestani Moghaddamfi et al. [31] provide valuable insights into faculty members' perspectives on observed stations, their impact on education, and possible areas for improvement. The study's findings highlight assessors' vital role in the assessment's educational effect. Students learning can be impacted by a plethora of factors, including the assessors' willingness to conduct a thorough assessment, their attitude toward the test, their comprehension of its goal, their experience and aptitude in creating a particular kind of exam, and their degree of leniency or strictness. Additionally, the research indicates that OSCE exams might help students connect newly learned material with existing knowledge, prolonging their learning. Regular evaluations are supposed to compound this impact. Students' experiences are also of utmost importance in this process. Numerous authors have noted the presence of examiner bias in clinical exam results [32].

Certain areas, such as "improved confidence in teaching clinical skills" and "improved confidence in giving students structured feedback," were also emphasized by thematic analysis of faculty’s perception of OSCE. These results align with prior research conducted by Sulaiman et al. on the Group Objective Structured Clinical Examination (GOSCE), which was administered to first-year, second-year, and third-year medical students. It was evident that the experience was appreciated by clinical instructors and students [33].

This study had several limitations: it was cross-sectional and conducted in a single department of a medical college. Another significant limitation of this study is the lack of a sample size calculation. This was due to practical challenges, such as restricted participant access and time limitations. Although this may impact the generalizability and statistical power of our results, it is worth noting that other studies in this field have encountered similar issues. Future research should incorporate a sample size calculation to improve the robustness and reliability of the findings. Therefore, generalizing the findings to other contexts should be done with caution. However, a notable strength was the feedback collection from both students and examiners, although the examiner feedback might be biased since they evaluated only one station.

## Conclusions

In this study, participants found the OSCE to be fair, well-structured, and thorough, covering essential knowledge areas. The OSCE stations were viewed as practical and valuable, with objective scoring. This finding reiterates that assessment tools should be reliable, valid, and transparent to gain greater acceptance among students and faculty. However, planning and implementing OSCEs requires considerable time and effort, which can be challenging for faculty. Additionally, the costs associated with OSCEs for large student groups can be high, though developing OSCE station banks and dedicated settings might help reduce expenses. Now is the ideal time to embed the OSCE into medical education as a core component of clinical skill assessment rather than just an additional method. Further research is needed to examine students’ experiences with OSCEs and to standardize their use in psychiatry. Additionally, multi-centric studies should be conducted to compare actual clinical performance between students assessed using traditional formats and those evaluated with OSCEs.
